# Analysis of Time Course and Dose Effect From Metformin on Body Mass Index in Children and Adolescents

**DOI:** 10.3389/fphar.2021.611480

**Published:** 2021-04-26

**Authors:** Dong-Dong Wang, Yi-Zhen Mao, Su-Mei He, Xiao Chen

**Affiliations:** ^1^ Jiangsu Key Laboratory of New Drug Research and Clinical Pharmacy, School of Pharmacy, Xuzhou Medical University, Xuzhou, China; ^2^ Department of Endocrinology, Xuzhou Central Hospital, Xuzhou, China; ^3^ Department of Pharmacy, The Affiliated Suzhou Science and Technology Town Hospital of Nanjing Medical University, Suzhou, China; ^4^ Department of Pharmacy, The People’s Hospital of Jiangyin, Jiangyin, China; ^5^ Department of Pharmacy, Children’s Hospital of Fudan University, Shanghai, China

**Keywords:** time course, dose effect, metformin, body mass index, children and adolescents

## Abstract

The purpose of this study was to analyze the time course and dose effect from metformin on body mass index (BMI) in children and adolescents by model-based meta-analysis (MBMA). Searching randomized controlled trial (RCT) studies of metformin on BMI in children and adolescents. The change rates of BMI from baseline values were used as indicator of evaluating metformin efficacy. A total of 18 RCT studies, 1,228 children and adolescents, were included for analysis, including patients with obesity, patients with type 1 diabetes mellitus, patients with nonalcoholic fatty liver, and patients with precocity. In order to achieve better effect of metformin on BMI in children and adolescents, the present study recommended that for patients with obesity, 1,000 mg/day metformin was required for at least 15.2 weeks and 60.8 weeks to achieve the plateau of metformin effect; for patients with type 1 diabetes mellitus, 1,000 mg/day metformin was required for at least 25.2 weeks and 100.8 weeks to achieve the plateau of metformin effect; for patients with nonalcoholic fatty liver, 1,000 mg/day metformin was required for at least 6.57 weeks and 26.28 weeks to achieve the plateau of metformin effect; for patients with precocity, 425 mg/day metformin was required for at least 12.4 weeks and 49.6 weeks to achieve the plateau of metformin effect. It was the first time to analyze the time course and dose effect from metformin on BMI and to recommend dosage and duration of treatment for metformin in children and adolescents with different disease types.

## Introduction

Metformin was widely used in the control of weight in different diseases of children and adolescents (Kendall et al., 2013; Pastor-Villaescusa et al., 2017). For example, patients with obesity (Atabek and Pirgon, 2008; Burgert et al., 2008; Love-Osborne et al., 2008; Clarson et al., 2009; Wilson et al., 2010; Yanovski et al., 2011; Gomez-Diaz et al., 2012; Kendall et al., 2013; van der Aa et al., 2016; Garibay-Nieto et al., 2017; Pastor-Villaescusa et al., 2017), patients with type 1 diabetes mellitus (Codner et al., 2013; Nadeau et al., 2015; Nwosu et al., 2015), patients with nonalcoholic fatty liver (Nadeau et al., 2009; Lavine et al., 2011), and patients with precocity (Ibanez et al., 2006a; Ibanez et al., 2006b). Metformin controlled weight mainly through decreasing caloric intake, including direct and indirect impacts on appetite regulation from the gastrointestinal side effects (Yerevanian and Soukas, 2019). In addition, metformin increased the peptide hormone growth/differentiation factor 15 (GDF15) circulating levels, lowering food intake and reducing body weight by means of the brain-stem–restricted receptor (Coll et al., 2020). However, the weight control of patients with metformin was off-label, lacking clinical dosage and duration of treatment recommendation. Especially, the time course and dose effect of metformin on body mass index (BMI) in children and adolescents with different disease types were unclear.

Model-based meta-analysis (MBMA) was a new quantitative pharmacological tool and could quantify dose course and time effect of drug (Mould, 2012; Wu et al., 2017). In particular, it could implement accurate pharmacodynamic comparison from the same drug in different disease types (Dong et al., 2018) and realize clinical drug dosage and duration of treatment recommendation (Chen et al., 2020a; Chen et al., 2020b; Cheng et al., 2020). The purpose of this study was to analyze the time course and dose effect from metformin on BMI using MBMA method and to recommend dosage and duration of treatment for metformin in children and adolescents with different disease types.

## Methods

### Literature Search and Data Extraction

We retrieved the Pubmed database (https://pubmed.ncbi.nlm.nih.gov/) with the deadline of September 2020. Only English publications were searched. Inclusion criteria included: 1) children and adolescents, 2) metformin treatment, 3) randomized controlled trial (RCT), 4) placebo controlled trial, and 5) with BMI information. Disease type, source, grouping, metformin dosage and duration of treatment, BMI, number of patient, and age were extracted from the above included studies. History and search details were shown in the Supporting Material (Supplementary Table S1). In order to eliminate the potential baseline effect, metformin efficacy (E) was evaluated using BMI change rate from baseline values in the present research. Eq. 1 was as follows:

$$\text{E\% }=\;\frac{{\text{E}}_{\text{t}}-{\text{E}}_{\text{b}}}{{\text{E}}_{\text{b}}}\;\times \;100\text{\%,}$$ (1)

E_t_, the value of BMI at time t; E_b_, the value of BMI at baseline.

### Model Establishment

In order to get actual drug effect on BMI from metformin, the placebo effect should be deducted from the metformin group. In addition, E_max_ models were used to assess the effect of metformin on BMI in children and adolescents with different disease types because the effect on BMI from metformin varied with time and reached a plateau. Eqs. 2, 3 were as follows:

$${\text{E}}_{\text{m, i, j}}{\;=\text{ E}}_{\text{g, i, j}}\;-{\text{E}}_{\text{p, i, j}},$$ (2)

$${\text{E}}_{\text{m, i, j}}\;=\;\frac{{\text{E}}_{\text{max},\text{ i},\text{ j }}\;\times \text{Time}}{{\text{ET}}_{50,\text{ i},\text{ j }}+\text{ Time}}+\frac{\varepsilon_{\text{i},\text{ j}}}{\sqrt{\frac{{\text{N}}_{\;\text{i},\text{j}}}{100}}},$$ (3)

E_g, i, j_, the sum effect on BMI from metformin, including actual metformin effect and placebo effect; E_m, i, j_, the actual metformin effect on BMI; E_p, i, j_, the placebo effect on BMI. i, different studies; j, time point of every study. E_max_, the maximal effect of metformin on BMI; ET_50_, the treatment duration to reach half of the maximal effect of metformin on BMI; *ε*_i, j_, the residual error of study i with j time; N_i, j_, the sample size in study i with time point j. *ε*_i, j_ was weighted by sample size, assumed to be normally distributed, with a mean of 0 and variance of *σ* ^2^/(N_i,j_/100).

Additive error or exponential error models were used to describe the inter-study variability. Eqs. 4–7 were as follows:

$${\text{E}}_{\text{max, i, j}}{\;=\text{ E}}_{\text{max}}\;+\;\eta_{1\text{,i}},$$ (4)

$${\text{ET}}_{50\text{, i, j }}{=\text{ ET}}_{50}\;+\;\eta_{2\text{,i}},$$ (5)

$${\text{E}}_{\text{max, i, j}}{\;=\text{ E}}_{\text{max}}\;\times \text{ exp }\left(\eta_{1\text{,i}}\right),$$ (6)

$${\text{ET}}_{50\text{, i, j}}{\;=\text{ ET}}_{50}\;\times \text{ exp }\left(\eta_{2\text{,i}}\right),$$ (7)

*η*_1,i_ and *η*_2,i_ were the inter-study variabilities; when available, they would be added into E_max_ and ET_50_, respectively. *η*_1,i_ and *η*_2,i_ were assumed to normally distributed, with a mean of 0 and variance of *ω*_1,i_^2^ and *ω*_2,i_^2^, respectively.

When building the covariates models, categorical covariates and continuous covariates were evaluated by Eqs. 8–10:

$${\text{P}}_{\text{p}}{\;=\text{ P}}_{\text{T}}\;+\text{ COV }\times \;\theta_{\text{c}},$$ (8)

$${\text{P}}_{\text{p}}{\;=\text{ P}}_{\text{T}}\;+\;\left({\text{COV }-\text{ COV}}_{\text{m}}\right)\;\times \theta_{\text{c}},$$ (9)

$${\text{P}}_{\text{p}}{\;=\text{ P}}_{\text{T}}\;\times \;{\left(\text{COV}/{\text{COV}}_{\text{m}}\right)}^{\theta_{\text{c}}},$$ (10)

COV, covariate; P_p_, the parameter for a patient with a covariate value of COV; P_T_, the typical value of the parameter; COV_m_, the median value of covariable in the population. *θ*_c_, a correction coefficient of the covariate to the model parameter.

The nonlinear mixed effect modeling (NONMEM, edition 7, ICON Development Solutions, Ellicott City, MD, United States) was used to build models. After a basic model was built, potential covariates were considered to add into E_max_. Objective function value (OFV) changes were as the covariate inclusion criteria. The decrease from OFV was greater than 3.84 (χ^2^, *α =* 0.05, d.f. = 1), which was considered sufficient for inclusion; the increase of OFV was greater than 6.63 (χ^2^, *α =* 0.01, d.f. = 1), which was considered sufficient for significance in the final model.

### Model Validation and Prediction

The final model accuracy was evaluated by visual inspection of routine diagnostic plots (individual predictions vs. observations). Prediction-corrected visual predictive check plots were used to assess the predictive performance of final models. The efficacy prediction from metformin on BMI in children and adolescents with different disease types was simulated by Monte Carlo method.

## Results

### Included Studies

**FIGURE 1:**
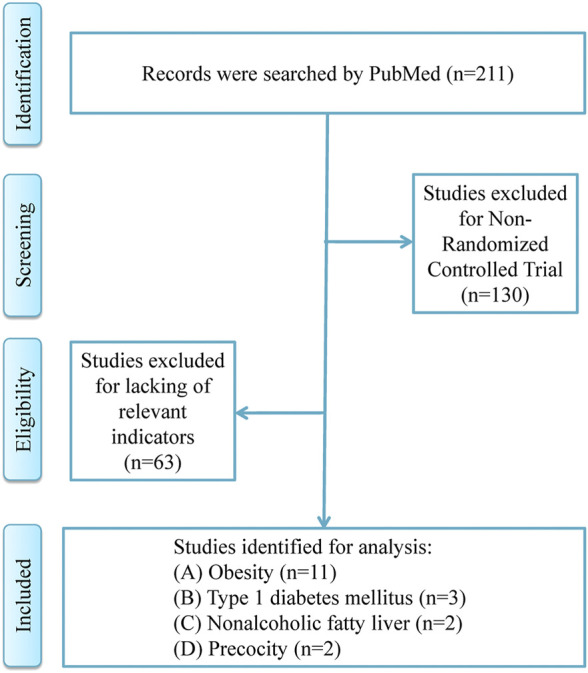
Strategy for literature search.

The process of literature search and details of included studies were shown in Figure 1; Table 1, respectively. A total of 18 RCT studies, 1,228 children and adolescents, were included for analysis, in which 11 studies for patients with obesity (Atabek and Pirgon, 2008; Burgert et al., 2008; Love-Osborne et al., 2008; Clarson et al., 2009; Wilson et al., 2010; Yanovski et al., 2011; Gomez-Diaz et al., 2012; Kendall et al., 2013; van der Aa et al., 2016; Garibay-Nieto et al., 2017; Pastor-Villaescusa et al., 2017), 3 studies for patients with type 1 diabetes mellitus (Codner et al., 2013; Nadeau et al., 2015; Nwosu et al., 2015), 2 studies for patients with nonalcoholic fatty liver (Nadeau et al., 2009; Lavine et al., 2011), and 2 studies for patients with precocity (Ibanez et al., 2006a; Ibanez et al., 2006b). In addition, in the included studies, the dosage ranges of metformin in patients with obesity were 1,000 mg/day–2,000 mg/day, in patients with type 1 diabetes mellitus were 1,000 mg/day–1,700 mg/day, in patients with nonalcoholic fatty liver were 1,000 mg/day–1,700 mg/day, and in patients with precocity were 425 mg/day–850 mg/day, respectively.

**TABLE 1:** Studies identified for analysis.

Disease	Study	Country	Group	Dosages (g/day)	Duration of treatment	Body mass index (kg/m^2^)	Number of people	Age (years)
Obesity	Pastor-Villaescusa et al. (2017)	Spain	Metformin (prepubertal)	1	6 months	28.2	40	6.8–15.3
			Placebo (prepubertal)		6 months	29.2	40	6.8–15.3
			Metformin (pubertal)	1	6 months	29.4	40	6.8–15.3
			Placebo (pubertal)		6 months	30.6	40	6.8–15.3
Obesity	Garibay-Nieto et al. (2017)	Mexico	Metformin	1	4 months	28.54	14	11.43 ± 2.1
			Placebo		4 months	28.79	17	12.59 ± 2.62
Obesity	van der Aa et al. (2016)	Netherlands	Metformin	2	18 months	29.8	23	12.6–15.3
			Placebo		18 months	30.5	19	11.3–14.0
Obesity	Kendall et al. (2013)	United Kingdom	Metformin	1.5	6 months	37.1	74	13.68 ± 2.3
			Placebo		6 months	35.95	77	13.64 ± 2.2
Obesity	Gómez-Díaz et al., (2012)	Mexico	Metformin	1.7	12 weeks	31.1	28	7.0–16.4
			Placebo		12 weeks	27.1	24	4.4–15.91
Obesity	Yanovski et al. (2011)	United States	Metformin	2	12 months	34.2	53	10.1 ± 1.6
			Placebo		12 months	34.6	47	10.4 ± 1.4
Obesity	Wilson et al. (2010)	United States	Metformin	2	52 weeks	35.9	39	14.8 ± 1.3
			Placebo		52 weeks	35.9	38	15.0 ± 1.5
Obesity	Clarson et al. (2009)	Canada	Metformin	1.5	6 months	36.4	11	10.1–16.1
			Placebo		6 months	33.9	14	10.1–16.1
Obesity	Burgert et al. (2008)	United States	Metformin	1.5	4 months	41	15	15 ± 2
			Placebo		4 months	40	13	15 ± 1
Obesity	Atabek and Pirgon(2008)	Turkey	Metformin	1	6 months	28.5	90	11.83 ± 2.8
			Placebo		6 months	28	30	11.6 ± 2.7
Obesity	Love-Osborne et al. (2008)	United States	Metformin	1.7	6 months	39.4	60	15.5 ± 1.7
			Placebo		6 months	39.3	25	14.2 ± 4.6
Type 1 diabetes mellitus	Nwosu et al. (2015)	United States	Metformin	1	9 months	28	15	15.0 ± 2.5
			Placebo		9 months	27.7	13	14.5 ± 3.1
Type 1 diabetes mellitus	Nadeau et al. (2015)	United States	Metformin	1	6 months	23.5	40	15.9 ± 1.7
			Placebo		6 months	24.3	40	16.0 ± 1.6
Type 1 diabetes mellitus	Codner et al. (2013)	Chile	Metformin	1.7	9 months	23.7	13	17.7 ± 1.6
			Placebo		9 months	26.2	11	16.7 ± 1.7
Nonalcoholic fatty liver	Lavine et al. (2011)	United States	Metformin	1	96 weeks	34	57	13.1 ± 2.4
			Placebo		96 weeks	33	58	12.9 ± 2.6
Nonalcoholic fatty liver	Nadeau et al. (2009)	United States	Metformin	1.7	6 months	39.6	37	12–18
			Placebo		6 months	40.2	13	12–18
Precocity	Ibáñez et al. (2006a)	Spain	Metformin	0.425	24 months	18.7	19	7.9 ± 0.2
			Placebo		24 months	18.1	19	8.0 ± 0.2
Precocity	Ibáñez et al. (2006b)	Spain	Metformin	0.85	36 months	21	10	9.0 ± 0.1
			Placebo		36 months	20.2	12	9.1 ± 0.1

### Modeling

The actual drug effect on BMI from metformin in children and adolescents with different disease types was shown in Table 2; the E_max_ of metformin on BMI in patients with obesity, patients with type 1 diabetes mellitus, patients with nonalcoholic fatty liver, and patients with precocity were -10, -4.31, -4.7, and -9.41%, respectively. The ET_50_ of metformin on BMI in patients with obesity, patients with type 1 diabetes mellitus, patients with nonalcoholic fatty liver, and patients with precocity were 15.2, 25.2, 6.57, and 12.4 weeks, respectively. In addition, no covariate (in particular dosage) was incorporated into the E_max_ models, showing there was no significant dose dependence from metformin efficacy on BMI in children and adolescents with different disease types from the current included studies.

**TABLE 2:** Parameter estimates of final models.

Model	Parameter	Estimate
(A)	E_max_, %	−10
	ET_50_, week	15.2
	ω_Emax_	6.025
	ω_ET50_	—
	ε	0.27
(C)	E_max_, %	−4.7
	ET_50_, week	6.57
	ω_Emax_	2.062
	ω_ET50_	—
	ε	0.01
(B)	E_max_, %	−4.31
	ET_50_, week	25.2
	*ω*_Emax_	1.995
	*ω*_ET50_	—
	ε	2.296
(D)	E_max_, %	−9.41
	ET_50_, week	12.4
	*ω*_Emax_	0.742
	*ω*_ET50_	—
	ε	0.01

The E_max_ models of metformin on BMI in patients with obesity, patients with type 1 diabetes mellitus, patients with nonalcoholic fatty liver, and patients with precocity were shown in Eqs. 11–14, respectively:

$$\text{E }=\;\frac{-10\text{\% }\times \text{Time}}{15.2+\text{ Time}},$$ (11)

$$\text{E }=\;\frac{-4.31\text{\% }\times \text{Time}}{25.2+\text{ Time}},$$ (12)

$$\text{E }=\;\frac{-4.7\text{\% }\times \text{Time}}{6.57+\text{ Time}},$$ (13)

$$\text{E }=\;\frac{-9.41\text{\% }\times \text{Time}}{12.4+\text{ Time}},$$ (14)

E, efficacy of metformin on BMI; Time, metformin treatment duration.

### Validation

**FIGURE 2:**
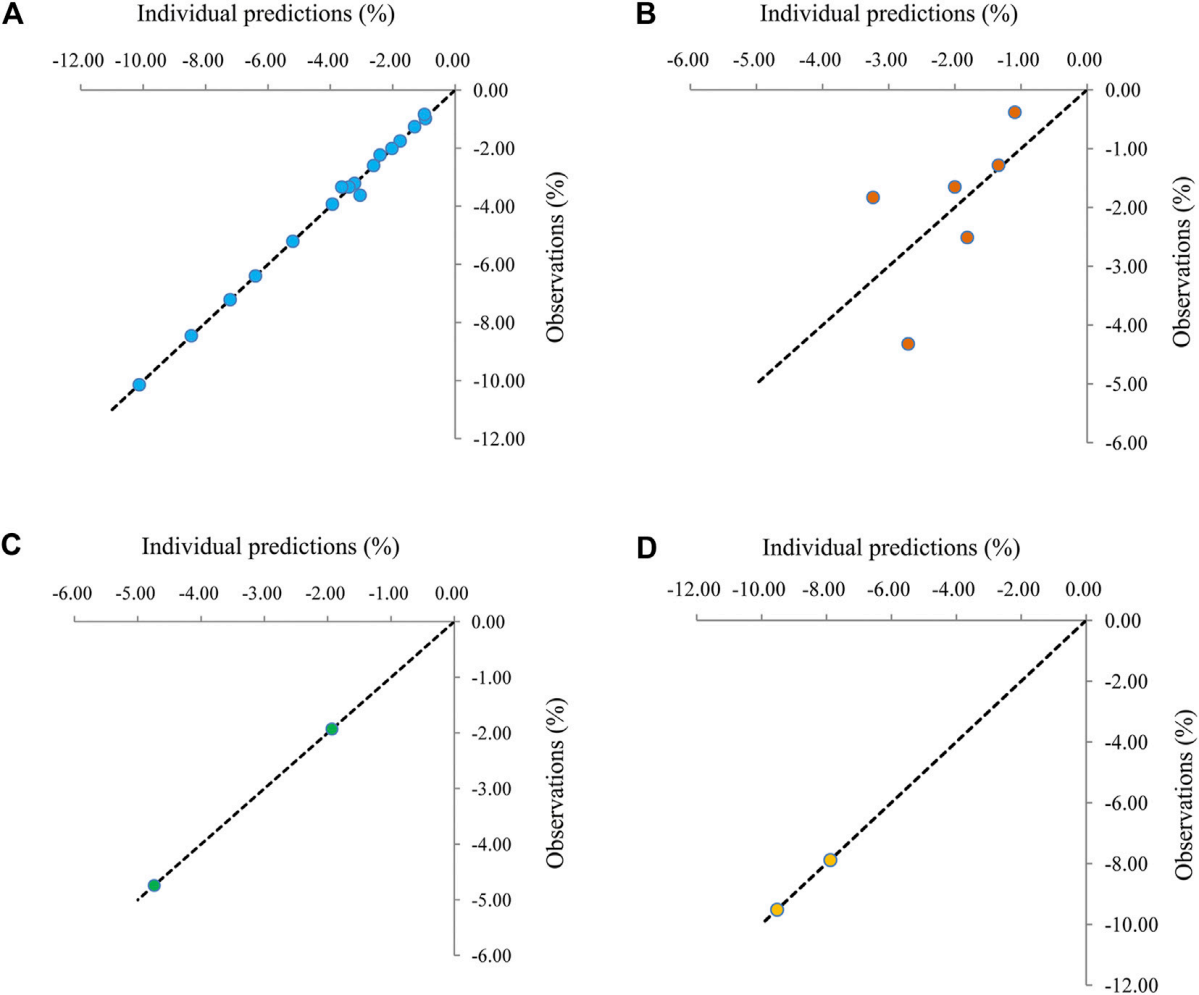
Visual inspection of routine diagnostic plots. **(A)** Patients with obesity, **(B)** patients with type 1 diabetes mellitus, **(C)** patients with nonalcoholic fatty liver., **(D)** patients with precocity.

The visual inspection of routine diagnostic plots was shown in Figure 2. Figures 2A–D were used to assess the final models of metformin on BMI in patients with obesity, patients with type 1 diabetes mellitus, patients with nonalcoholic fatty liver, and patients with precocity, respectively. As we could see, there were good linear relationships between individual predictions and observations, meaning the good fitting of the final models.

**FIGURE 3:**
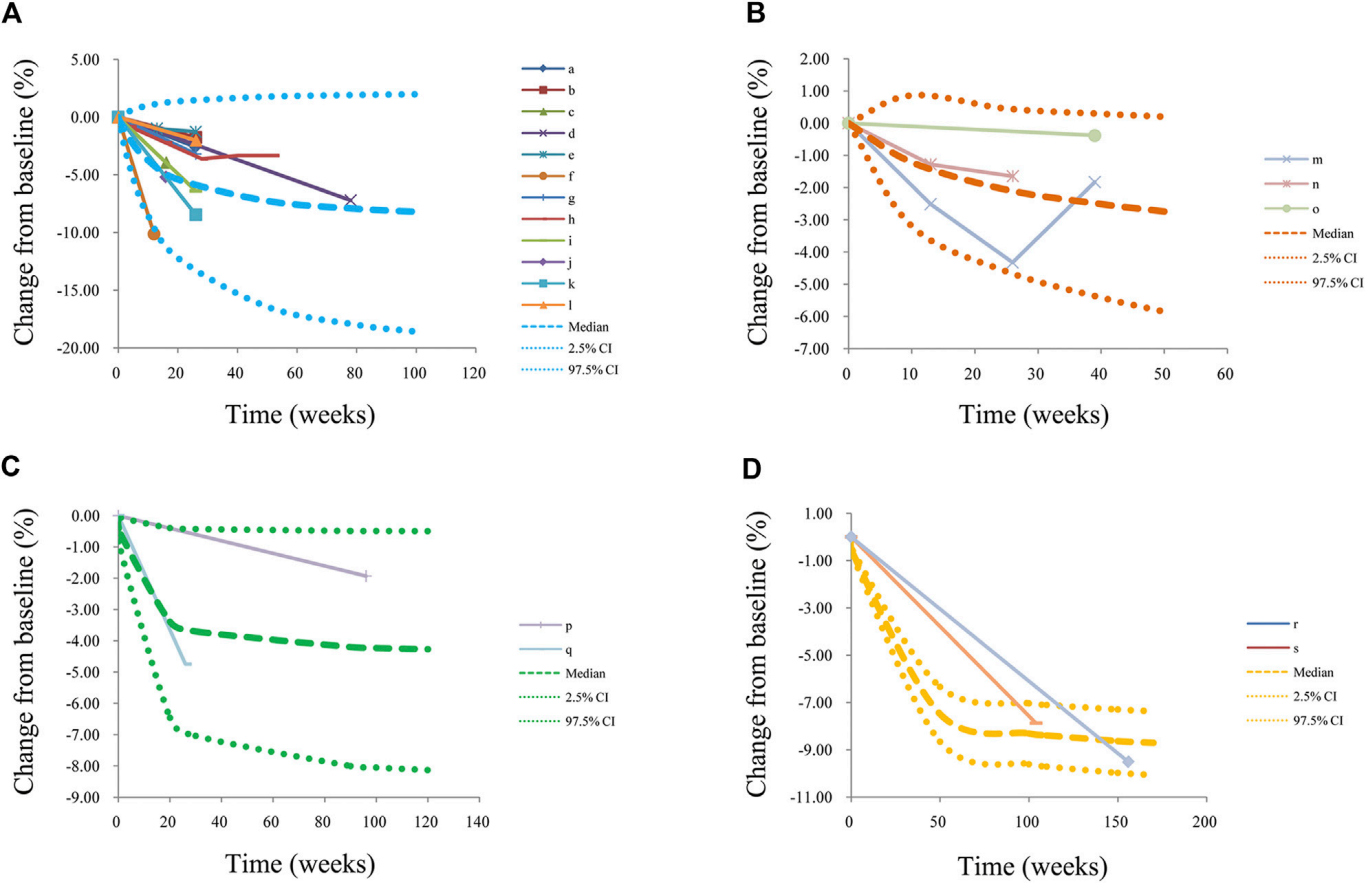
Prediction-corrected visual predictive check plots. **(A)** patients with obesity, **(B)** patients with type 1 diabetes mellitus, **(C)** patients with nonalcoholic fatty liver, and **(D)** patients with precocity. Median, 2.5% CI, and 97.5% CI were simulated by Monte Carlo (n = 1,000); CI; a, Pastor-Villaescusa et al. (2017); b, Pastor-Villaescusa et al. (2017); c, Garibay-Nieto et al. (2017); d, van der Aa et al. (2016); e, Kendall et al. (2013); f, Gómez-Díaz et al. (2012); g, Yanovski et al. (2011); h, Wilson et al. (2010); i, Clarson et al. (2009); j, Burgert et al. (2008); k, Atabek and Pirgon (2008); l, Love-Osborne et al. (2008); m, Nwosu et al. (2015); n, Nadeau et al. (2015); o, Codner et al. (2013); p, Lavine et al. (2011); q, Nadeau et al. (2009); r, Ibáñez et al. (2006a); s, Ibáñez et al. (2006b).

The visual predictive check plots were shown in Figure 3. Figures 3A–D, were used to evaluate the predictive performance of final models from metformin on BMI in patients with obesity, patients with type 1 diabetes mellitus, patients with nonalcoholic fatty liver, and patients with precocity, respectively, in which most observed data were included in the 95% prediction intervals produced by simulation data, showing the predictive power of the final models.

### Prediction

**FIGURE 4:**
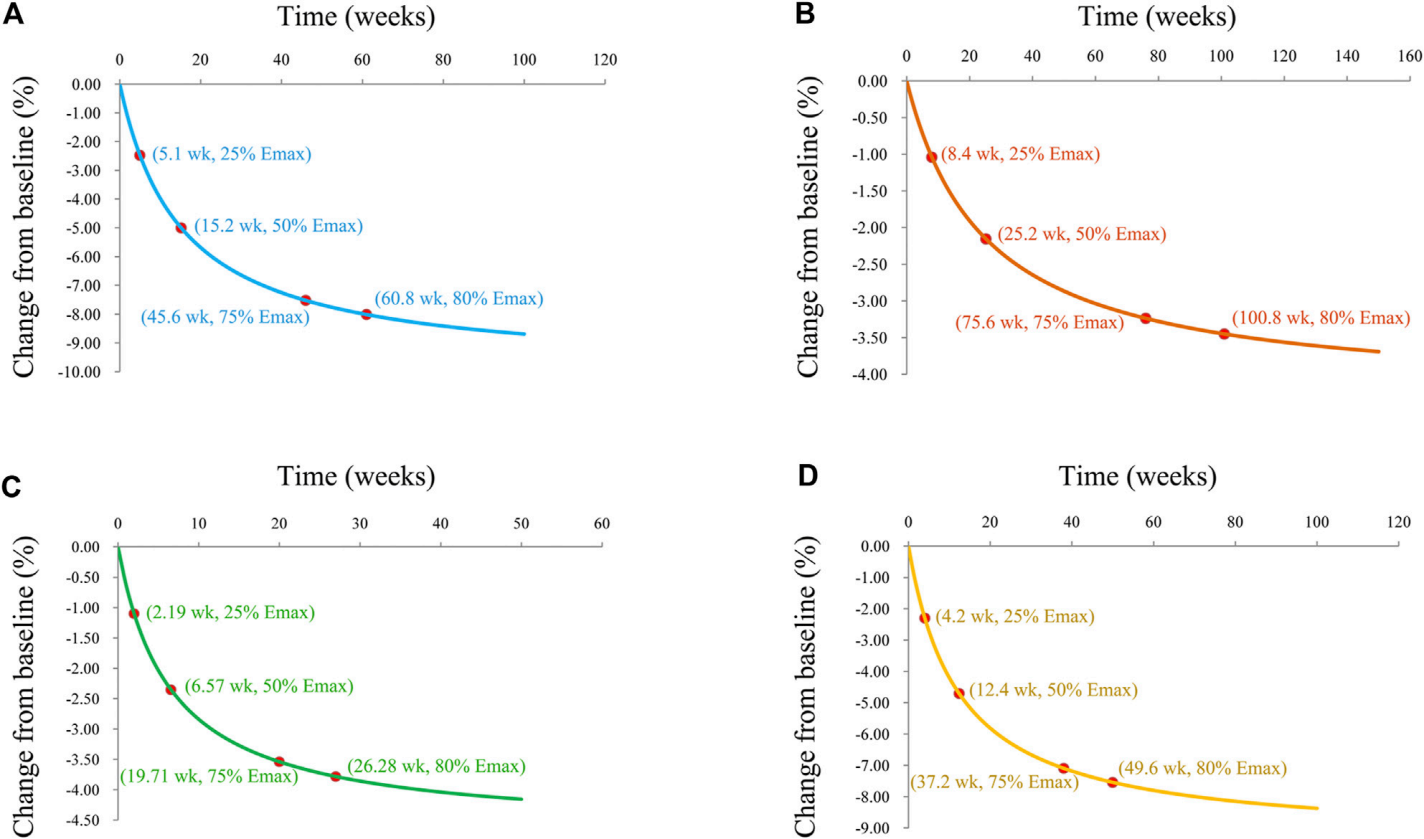
Model prediction. **(A)** Patients with obesity, **(B)** patients with type 1 diabetes mellitus, **(C)** patients with nonalcoholic fatty liver, and **(D)** patients with precocity.

The trends of efficacy of metformin on BMI in patients with obesity, patients with type 1 diabetes mellitus, patients with nonalcoholic fatty liver, and patients with precocity were shown in Figures 4A–D, respectively. In patients with obesity, the efficacy of metformin on BMI at 5.1 weeks was 25% of the E_max_, at 15.2 weeks was 50% of the E_max_, at 45.6 weeks was 75% of the E_max_, and at 60.8 weeks was 80% of the E_max_. In patients with type 1 diabetes mellitus, the efficacy of metformin on BMI at 8.4 weeks was 25% of the E_max_, at 25.2 weeks was 50% of the E_max_, at 75.6 weeks was 75% of the E_max_, and at 100.8 weeks was 80% of the E_max_. In patients with nonalcoholic fatty liver, the efficacy of metformin on BMI at 2.19 weeks was 25% of the E_max_, at 6.57 weeks was 50% of the E_max_, at 19.71 weeks was 75% of the E_max_, and at 26.28 weeks was 80% of the E_max_. In patients with precocity, the efficacy of metformin on BMI at 4.2 weeks was 25% of the E_max_, at 12.4 weeks was 50% of the E_max_, at 37.2 weeks was 75% of the E_max_, and at 49.6 weeks was 80% of the E_max_.

## Disscusion

Metformin was increasingly used to control weight in children and adolescents, such as patients with obesity (Atabek and Pirgon, 2008; Burgert et al., 2008; Love-Osborne et al., 2008; Clarson et al., 2009; Wilson et al., 2010; Yanovski et al., 2011; Gomez-Diaz et al., 2012; Kendall et al., 2013; van der Aa et al., 2016; Garibay-Nieto et al., 2017; Pastor-Villaescusa et al., 2017), patients with type 1 diabetes mellitus (Codner et al., 2013; Nadeau et al., 2015; Nwosu et al., 2015), patients with nonalcoholic fatty liver (Nadeau et al., 2009; Lavine et al., 2011), and patients with precocity (Ibanez et al., 2006a; Ibanez et al., 2006b). However, lacking dosage and duration of treatment recommendation made it difficult to promote metformin usage in children and adolescents with different disease types for control of weight. The present study was to analyze the time course and dose effect from metformin on BMI using the MBMA method and to recommend dosage and duration of treatment from metformin in children and adolescents with different disease types.

In the present study, the E_max_ of metformin on BMI in patients with obesity, patients with type 1 diabetes mellitus, patients with nonalcoholic fatty liver, and patients with precocity were -10, -4.31, -4.7, and -9.41, respectively. The ET_50_ of metformin on BMI in patients with obesity, patients with type 1 diabetes mellitus, patients with nonalcoholic fatty liver, and patients with precocity were 15.2, 25.2, 6.57, and 12.4 weeks, respectively. Of course, the present study was not isolated. In the previous study from Chen *et al*, the change rate of weight from baseline was selected as the efficacy indicator, and the ET50 in patients with obesity was 15.1 weeks (Chen et al., 2020b), which had the similar ET_50_ value with the present study. However, the population in the previous study was not entirely children and adolescents, and the number of previously included studies in Chen et al. (2020b) was also lower than that of the current study.

In addition, in our present study, no covariate (in particular, metformin dosage) was incorporated into the E_max_ models, showing there was no significant dose dependence from metformin efficacy on BMI in children and adolescents with different disease types from the current included studies. In other words, we could recommend the lower limits of the metformin dose ranges on the basis of the current included studies for different disease types. Further, to achieve the 80% efficacy of E_max_, which was called plateau, metformin treatment duration were 60.8, 100.8, 26.28, and 49.6 weeks in patients with obesity, patients with type 1 diabetes mellitus, patients with nonalcoholic fatty liver, and patients with precocity, respectively. For type 1 diabetes mellitus, no study went beyond 9 months, yet estimates for metformin effect were being extrapolated to 100.8 weeks, which would be further verified in the future research. Furthermore, countries were also analyzed in this study as a categorical covariable, however, were not included in the E_max_ models, revealing there was no significant country dependence from metformin efficacy on BMI in children and adolescents with different disease types from the current included studies.

The present study recommended that for patients with obesity, 1,000 mg/day metformin was required for at least 15.2 weeks, and 60.8 weeks to achieve the plateau of metformin effect. However, in the study from Pu et al (2020), effects of metformin in obesity treatment, they found high dose metformin (>1,500 mg/d) was more effective in reducing BMI, yet more than half of their studies were on adults. In our study, only children and adolescents were involved. That was to say, the composition of the population would have a certain impact on the choice of dosage. The optimal dose for children and adolescents was lower than that for adults. In addition, for patients with type 1 diabetes mellitus, 1,000 mg/day metformin was required for at least 25.2 weeks and 100.8 weeks to achieve the plateau of metformin effect. For patients with nonalcoholic fatty liver, 1,000 mg/day metformin was required for at least 6.57 weeks and 26.28 weeks to achieve the plateau of metformin effect. For patients with precocity, 425 mg/day metformin was required for at least 12.4 weeks and 49.6 weeks to achieve the plateau of metformin effect.

However, there were limited data for all the various dosages studied across the timeframe; for nonalcoholic fatty liver and precocity, only 2 studies were identified. In addition, there were almost no studies on children younger than 4 years old included in the present study. In terms of geographical scope, the Asian countries and populations involved in this study were small. Therefore, the results of this study would be further verified in the following clinical studies or trials.

## Conclusion

It was the first time to analyze the time course and dose effect from metformin on BMI in children and adolescents and meanwhile to recommend dosage and duration of treatment for metformin in patients with obesity, patients with type 1 diabetes mellitus, patients with nonalcoholic fatty liver, and patients with precocity, respectively. The recommendations would be validated in future clinical studies or trials.

## Data Availability

The original contributions presented in the study are included in the article/Supplementary Material, and further inquiries can be directed to the corresponding authors.
