# Supplementary material for: Analysis of Time Course and Dose Effect From Metformin on Body Mass Index in Children and Adolescents
Source: Front Pharmacol. 2021 Apr 26;12:611480. doi: 10.3389/fphar.2021.611480 (PMC8107689; doi:10.3389/fphar.2021.611480)
Supplement: Supplementary file 1 [file table1.docx]

**Table S1. Search details**

| No. | Query | Results |
| --- | --- | --- |
| #1 | Metformin[Title] | 10,822 |
| #2 | Dimethyldiguanide[Title] | 6 |
| #3 | Glucophage[Title] | 38 |
| #4 | Child[Title] | 119,067 |
| #5 | Children[Title] | 531,931 |
| #6 | Paediatric[Title] | 24,317 |
| #7 | Pediatric[Title] | 132,261 |
| #8 | Pediatrics[Title] | 11,758 |
| #9 | Boys[Title] | 8,864 |
| #10 | Girls[Title] | 13,572 |
| #11 | Adolescent[Title] | 57,125 |
| #12 | Adolescents[Title] | 94,053 |
| #13 | Youth[Title] | 27,122 |
| #14 | Young People[Title] | 7,725 |
| #15 | Puberty[Title] | 8,190 |
| #16 | Pubertal[Title] | 3,974 |
| #17 | #1 OR #2 OR #3 | 10,856 |
| #18 | #4 OR #5 OR #6 OR #7 OR #8 OR #9 OR#10 OR #11 OR #12 OR #13 OR #14 OR #15 OR #16 | 968,300 |
| #19 | #17 AND #18 | 211 |
| #20 | #19 Filters: Randomized Controlled Trial | 81 |

**List of final included studies:**

**(A) Patients with obesity (n=11)**

1. Pastor-Villaescusa B, Cañete MD, Caballero-Villarraso J, et al. Metformin for Obesity in Prepubertal and Pubertal Children: A Randomized Controlled Trial [published correction appears in Pediatrics. 2017 Nov;140(5):] [published correction appears in Pediatrics. 2017 Nov 30;:]. *Pediatrics*. 2017;140(1):e20164285. doi:10.1542/peds.2016-4285

2. Garibay-Nieto N, Queipo-García G, Alvarez F, et al. Effects of Conjugated Linoleic Acid and Metformin on Insulin Sensitivity in Obese Children: Randomized Clinical Trial. *J Clin Endocrinol Metab*. 2017;102(1):132-140. doi:10.1210/jc.2016-2701

3. van der Aa MP, Elst MA, van de Garde EM, van Mil EG, Knibbe CA, van der Vorst MM. Long-term treatment with metformin in obese, insulin-resistant adolescents: results of a randomized double-blinded placebo-controlled trial. *Nutr Diabetes*. 2016;6(8):e228. Published 2016 Aug 29. doi:10.1038/nutd.2016.37

4. Kendall D, Vail A, Amin R, et al. Metformin in obese children and adolescents: the MOCA trial. *J Clin Endocrinol Metab*. 2013;98(1):322-329. doi:10.1210/jc.2012-2710

5. Gómez-Díaz RA, Talavera JO, Pool EC, et al. Metformin decreases plasma resistin concentrations in pediatric patients with impaired glucose tolerance: a placebo-controlled randomized clinical trial. *Metabolism*. 2012;61(9):1247-1255. doi:10.1016/j.metabol.2012.02.003

6. Yanovski JA, Krakoff J, Salaita CG, et al. Effects of metformin on body weight and body composition in obese insulin-resistant children: a randomized clinical trial. *Diabetes*. 2011;60(2):477-485. doi:10.2337/db10-1185

7. Wilson DM, Abrams SH, Aye T, et al. Metformin extended release treatment of adolescent obesity: a 48-week randomized, double-blind, placebo-controlled trial with 48-week follow-up. *Arch Pediatr Adolesc Med*. 2010;164(2):116-123. doi:10.1001/archpediatrics.2009.264

8. Clarson CL, Mahmud FH, Baker JE, et al. Metformin in combination with structured lifestyle intervention improved body mass index in obese adolescents, but did not improve insulin resistance. *Endocrine*. 2009;36(1):141-146. doi:10.1007/s12020-009-9196-9

9. Burgert TS, Duran EJ, Goldberg-Gell R, et al. Short-term metabolic and cardiovascular effects of metformin in markedly obese adolescents with normal glucose tolerance. *Pediatr Diabetes*. 2008;9(6):567-576. doi:10.1111/j.1399-5448.2008.00434.x

10. Atabek ME, Pirgon O. Use of metformin in obese adolescents with hyperinsulinemia: a 6-month, randomized, double-blind, placebo-controlled clinical trial. *J Pediatr Endocrinol Metab*. 2008;21(4):339-348. doi:10.1515/jpem.2008.21.4.339

11. Love-Osborne K, Sheeder J, Zeitler P. Addition of metformin to a lifestyle modification program in adolescents with insulin resistance. *J Pediatr*. 2008;152(6):817-822. doi:10.1016/j.jpeds.2008.01.018

**(B) Patients with type 1 diabetes mellitus (n=3)**

12. Nwosu BU, Maranda L, Cullen K, et al. A Randomized, Double-Blind, Placebo-Controlled Trial of Adjunctive Metformin Therapy in Overweight/Obese Youth with Type 1 Diabetes. *PLoS One*. 2015;10(9):e0137525. Published 2015 Sep 14. doi:10.1371/journal.pone.0137525

13. Nadeau KJ, Chow K, Alam S, et al. Effects of low dose metformin in adolescents with type I diabetes mellitus: a randomized, double-blinded placebo-controlled study. *Pediatr Diabetes*. 2015;16(3):196-203. doi:10.1111/pedi.12140

14. Codner E, Iñíguez G, López P, et al. Metformin for the treatment of hyperandrogenism in adolescents with type 1 diabetes mellitus. *Horm Res Paediatr*. 2013;80(5):343-349. doi:10.1159/000355513

**(C) Patients with nonalcoholic fatty liver (n=2)**

15. Lavine JE, Schwimmer JB, Van Natta ML, et al. Effect of vitamin E or metformin for treatment of nonalcoholic fatty liver disease in children and adolescents: the TONIC randomized controlled trial. *JAMA*. 2011;305(16):1659-1668. doi:10.1001/jama.2011.520

16. Nadeau KJ, Ehlers LB, Zeitler PS, Love-Osborne K. Treatment of non-alcoholic fatty liver disease with metformin versus lifestyle intervention in insulin-resistant adolescents. *Pediatr Diabetes*. 2009;10(1):5-13. doi:10.1111/j.1399-5448.2008.00450.x

**(D) Patients with precocity (n=2)**

17. Ibáñez L, Ong K, Valls C, Marcos MV, Dunger DB, de Zegher F. Metformin treatment to prevent early puberty in girls with precocious pubarche. *J Clin Endocrinol Metab*. 2006;91(8):2888-2891. doi:10.1210/jc.2006-0336

18. Ibáñez L, Valls C, Ong K, Dunger DB, de Zegher F. Metformin therapy during puberty delays menarche, prolongs pubertal growth, and augments adult height: a randomized study in low-birth-weight girls with early-normal onset of puberty. *J Clin Endocrinol Metab*. 2006;91(6):2068-2073. doi:10.1210/jc.2005-2329
